# The crystalline sponge method updated

**DOI:** 10.1107/S2052252515024379

**Published:** 2016-02-12

**Authors:** Manabu Hoshino, Anupam Khutia, Hongzhu Xing, Yasuhide Inokuma, Makoto Fujita

**Affiliations:** a Department of Applied Chemistry, Graduate School of Engineering, The University of Tokyo, and ACCEL (JST), 7-3-1 Hongo, Bunkyo-ku, Tokyo 113-8656, Japan

**Keywords:** crystalline sponge method, porous materials, metal–organic frameworks, chemical crystallography

## Abstract

Crystalline sponges are porous metal complexes that can absorb and orient common organic molecules in their pores and make them observable by conventional X-ray structure analysis (crystalline sponge method). In this study, all of the steps in the crystalline sponge method, including sponge crystal preparation, pore–solvent exchange, guest soaking, data collection and crystallographic analysis, are carefully examined and thoroughly optimized to provide reliable and meaningful chemical information as chemical crystallography. Major improvements in the method have been made in the guest-soaking and data-collection steps. In the soaking step, obtaining a high site occupancy of the guest is particularly important, and dominant parameters for guest soaking (*e.g.* temperature, time, concentration, solvents) therefore have to be optimized for every sample compound. When standard conditions do not work, a high-throughput method is useful for efficiently optimizing the soaking conditions. The X-ray experiments are also carefully re-examined. Significant improvement of the guest data quality is achieved by complete data collection at high angle regions. The appropriate disorder treatment of the most flexible ZnI_2_ portions of the host framework and refinement of the solvents filling the remaining void are also particularly important for obtaining better data quality. A benchmark test for the crystalline sponge method toward an achiral molecule is proposed with a guaiazulene guest, in which the guest structure (with ∼ 100% site occupancy) is refined without applying any restraints or constraints. The obtained data quality with *R*_int_ = 0.0279 and *R*_1_ = 0.0379 is comparable with that of current conventional crystallographic analysis for small molecules. Another benchmark test for this method toward a chiral molecule is also proposed with a santonin guest. The crystallographic data obtained [*R*_int_ = 0.0421, *R*_1_ = 0.0312, Flack (Parsons) = −0.0071 (11)] represents the potential ability of this method for reliable absolute structure determination.

## Abstract 2

The protocols of the crystalline sponge method, particularly those in the soaking, data collection and refinement processes, are considerably improved to give reliable structural information.

## 1. Introduction   

**Figure 1:**
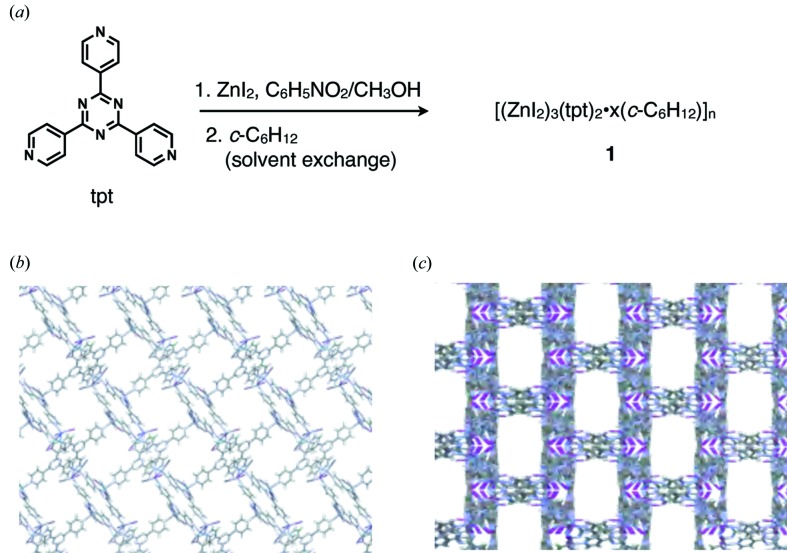
(*a*) Preparation of the most potent crystalline sponge [(ZnI_2_)_3_(tpt)_2_·*x*(solvent)]_*n*_ (**1**). (*b*, *c*) Packing views of **1** in the (*b*) [010] and (*c*) [101] directions. Solvent molecules (cyclohexane) filling the void space are omitted for clarity.

As the study of molecules, chemistry is concerned with uncovering the molecular structures of compounds of interest. X-ray crystallographic analysis is the only method that provides direct information on molecular structures at the atomic level. However, this method has the intrinsic limitation that the target molecules must be crystalline, and high quality single crystals must be prepared before measurement (Blake, 2009 ▸). This limitation has often been a considerable bottle-neck for the development of molecular chemistry, in particular when the structural determination of the target compound is the major concern of the research (*e.g.* structural determination of isolated natural products, total synthesis studies and product analysis in new reactions). In 2013, we reported a new technique for single-crystal X-ray diffraction analysis that does not require the crystallization of samples in the sample preparation (Inokuma *et al.*, 2013 ▸). This method, later called the crystalline sponge method (Vinogradova *et al.*, 2014 ▸), uses crystals of porous metal complexes (Hoskins & Robson, 1989 ▸; Fujita *et al.*, 1994 ▸; Subramanian & Zaworotko, 1995 ▸; Yaghi & Li, 1995 ▸; Kondo *et al.*, 1997 ▸; Mori *et al.*, 1997 ▸; Zhou & Kitagawa, 2014 ▸; Zhou *et al.*, 2012 ▸) [crystalline sponges (Biradha & Fujita, 2002 ▸; Inokuma *et al.*, 2010 ▸) Fig. 1 ▸] capable of absorbing guest compounds from solution in a common solvent. The guests are efficiently trapped and concentrated at several binding sites in the porous complexes, and the periodic array of the binding sites renders the absorbed guests oriented and observable by common X-ray diffraction studies (Kawano & Fujita, 2007 ▸; Biradha *et al.*, 2002 ▸; Suh *et al.*, 2002 ▸; Ohmori *et al.*, 2004 ▸; Ohmori *et al.*, 2005 ▸). Since only one tiny piece of crystal, weighing approximately 1 µg, is enough to perform the experiment, the required amount of the guest is on the nano-to-microgram order. Owing to the removal of the crystallization step from the sequential procedure of single-crystal X-ray experiments, considerable attention has been paid to the crystalline sponge method by the communities of organic synthetic chemists, natural product chemists, coordination chemists and crystallographers, as well as researchers in the pharmaceutical, agriculture and food industries. Following our report, the utility of the method has been independently demonstrated by several reports from other groups (Vinogradova *et al.*, 2014 ▸; Ramadhar *et al.*, 2015*a* ▸,*b* ▸; Sanna *et al.* 2015 ▸) in which the crystalline sponge method and related techniques were used to determine the structures of small molecules. Useful applications of this method for absolute structure determination (Yoshioka *et al.*, 2015 ▸) and metabolite analysis (Zigon *et al.*, 2015 ▸) have been recently reported by our group and others (Kamimura *et al.*, 2013 ▸; O’Brien *et al.*, 2014 ▸; Tashiro *et al.*, 2012 ▸; Kubota *et al.*, 2014 ▸; Turega *et al.*, 2014 ▸).

In our original report, however, the data quality of the trapped guest compounds was not very high and the use of restraints and constraints based on chemical information was necessary to refine the guest structures. This was not due to insufficient knowledge or techniques of crystallographic analysis but instead due to unoptimized experimental protocols throughout the method (Inokuma *et al.*, 2013 ▸); the data quality depends on all the steps in the crystalline sponge method, including the synthesis of high-quality sponges, solvent exchange, guest-soaking, data collection and crystallographic refinement of the post-absorbed molecules. These steps were largely unfamiliar to all researchers and the method was still at a proof-of-concept stage. To develop the crystalline sponge method from basic science into a reliable new technology that may innovate all of the molecular sciences, much effort is needed to be put into improving the data quality. In addition, the crystallographic scope and limitations in the refinement of structures with large pores [so-called metal–organic framework (MOF) structures] should be carefully considered (Spek, 2015 ▸). Over the last 2 years, therefore, our group has made considerable efforts to improve the data quality and to uncover the crystallographic scope and limitations for the refinement of guest structures obtained using the crystalline sponge method. In this article, we describe certain improved procedures in crystal soaking and crystallography of the crystalline sponge method, without which the method cannot be used by the general working chemist. The updated methods and protocols are applied to most of the compounds reported in our original paper (Inokuma *et al.*, 2013 ▸).

It is also important to understand that, unlike scientific principles, there are no clear right or best answers for the experimental protocols because they are highly dependent on the nature of the samples, the purpose of the analysis, and the skill or taste of the users. Recently, Clardy reported practical guidelines for our crystalline sponge method, in which some modified or improved procedures on our original method are recommended (Ramadhar *et al.*, 2015*a* ▸,*b* ▸). Similar attempts to improve the original protocols that may appear in a near future by other groups are welcome. The protocols discussed here, as well as those suggested by the others, are still tentative and they should be ideally elaborated through future discussions among users and by many successful results. Nevertheless, we also emphasize that, without the procedures outlined in this paper, the crystalline sponge method cannot be generally applied by bench chemists or crystallographers.

## 2. Results and discussion   

### 2.1. A standard protocol for sample preparation   

After the publication of the original paper, we also published a detailed experimental protocol for the crystalline sponge method (Inokuma *et al.*, 2013 ▸) using two standard samples, guaiazulene and 2,6-diisopropylaniline, particularly focusing on the guest-soaking step. Guaizulene is a suitable guest for the crystalline sponge method because the guest-soaking process proceeds relatively smoothly and guest inclusion is clearly indicated by the color change of the sponge crystal. The reported protocol was, however, optimized only for the two standard compounds and cannot necessarily be applied to a wide range of organic guest compounds. The soaking process must be optimized for every compound and is dependent on its molecular size, polarity, flexibility, nucleophilicity, stability, solubility and other properties. In summary, there is no universal protocol in the soaking step that can be applied to any compounds. The following discussion will be carried out based on this understanding.

Single crystal samples for the crystalline sponge method are prepared by the following steps: (1) host crystal synthesis, (2) solvent exchange and (3) inclusion of the target molecules to be analyzed. Although the detailed procedures in each step have been described in a previous paper (Inokuma *et al.*, 2013 ▸), we have here added some new or updated procedures that have improved the method in terms of data quality, practicability and applicability.

#### 2.1.1. [(ZnI_2_)_3_(tpt)_2_·*x*(solvent)]_*n*_ as a crystalline sponge   

**Figure 2:**
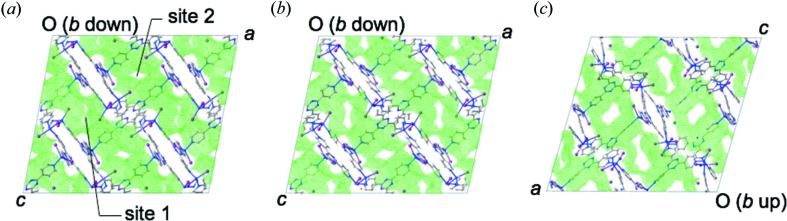
The crystal structures of crystalline sponges with the pores filled by (*a*) cyclohexane, (*b*) guaiazulene and (*c*) santonin. The wall of pores is presented with semi-transparent van der Waals surfaces colored in green. Guest-induced deformation of the pores is observed.

In our original paper, two porous complexes, [(ZnI_2_)_3_(tpt)_2_·*x*(solvent)]_*n*_ (**1**) (Biradha & Fujita, 2002 ▸) and [[Co(SCN)_2_]_3_(tpt)_2_·*x*(solvent)]_*n*_ (**2**) (Inokuma *et al.*, 2010 ▸) [tpt = 1,3,5-tris(4-pyridyl)triazine], were used as crystalline sponges. Regarding complex **2**, absorbed guest molecules were quite prone to static disorder because they frequently lay on the symmetry elements of the cubic lattice (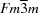). In terms of practicability, we are thus currently using the less symmetric (*C*2/*c*) complex **1** in most cases as the crystalline sponge. In addition to the relatively low symmetry of the space group, complex **1** is a suitable crystalline sponge because of the following advantages in host–guest complexation in the pores: (1) The size of the pores (5 × 8 Å measured between van der Waals surfaces in the cross section projected from the *b*-axis) is appropriate for the accommodation of organic molecules of common sizes. (2) The tpt ligand offers flat and electron-deficient binding sites suitable for stacking with aromatic compounds and for CH–π interactions even with aliphatic compounds (Ohmori *et al.*, 2004 ▸). (3) The cavity is rather hydrophobic and favorable for the binding of common organic molecules. (4) The I atoms in the ZnI_2_ component are good hydrogen-bond acceptors and the pyridyl protons of the tpt ligand are good hydrogen-bond donors, both providing efficient binding sites through hydrogen-bonding. (5) The framework is relatively flexible and guests are often bound in the cavity by induced-fit molecular recognition. Regarding the pore size (5 × 8 Å), it does not strictly limit the size of guest to be included because the sponge framework is very flexible and guest molecules larger than the portal are often accommodated by expanding the pore size. Typical expansion of the pores by inclusion of guaiazulene and santonin is visualized in Fig. 2 ▸.

**Figure 3:**
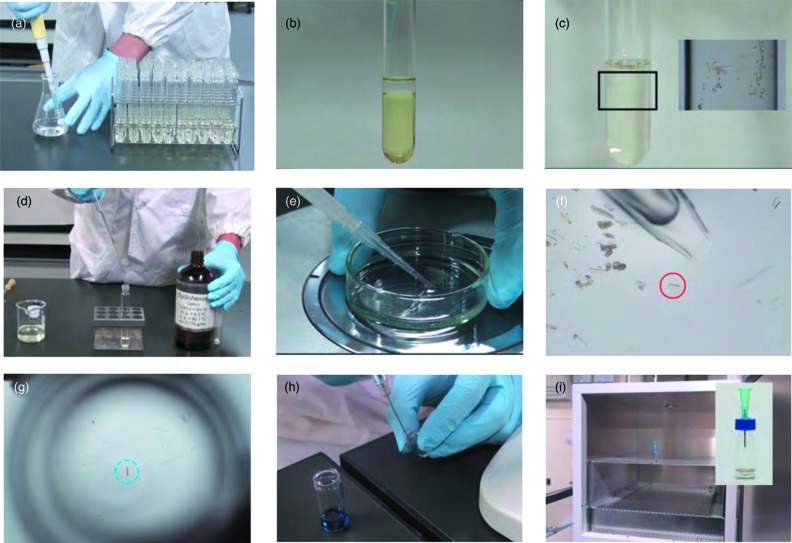
Instruction for the preparation of solvent-exchanged crystalline sponge (**1**) which is ready to use. (*a*, *b*) ZnI_2_/CH_3_OH solution is layered on tpt/nitrobenzene solution in a set of 50 test tubes. The two-layered mixture solution is allowed to stand for 1 week to obtain single crystals of as-synthesized **1**. (*c*) A picture of as-synthesized **1** formed in a test tube. (*d*) Crystals are washed with cyclohexane. (*e*, *f*) High quality crystalline sponges (rod-shaped, ∼ 100 µm-sized, and non-clacked) are chosen, transferred to vials, and (*g*) placed on the center of a microvial. (*h*) Guest compound (∼ 5 µg) is injected. (*i*) A needle is pierced for slow evaporation of the solvent, and the vial is kept in an incubator for guest-soaking.

As previously described in detail (Inokuma *et al.*, 2013 ▸), crystalline sponge **1** is synthesized by the layer diffusion of a methanolic solution of ZnI_2_ into a PhNO_2_/MeOH (*v*/*v* = 4/1) solution of the tpt ligand at room temperature in a test tube (Fig. 3 ▸). Isostructural ZnCl_2_ and ZnBr_2_ complexes, [(ZnCl_2_)_3_(tpt)_2_·*x*(solvent)]_*n*_ (Ramadhar *et al.*, 2015*a* ▸,*b* ▸; Batten & Robson, 1998 ▸; Heine *et al.*, 2011 ▸) and [(ZnBr_2_)_3_(tpt)_2_·*x*(solvent)]_*n*_ (Ramadhar *et al.*, 2015*a* ▸,*b* ▸; Kawano *et al.*, 2008 ▸) have been reported. These complexes are useful when the strong residual electron peaks around the heavy (iodine) atoms due to the termination effect from moderate data resolution obscure the weak peaks of the nearby guests.

#### 2.1.2. What determines the data quality?   

Unlike common X-ray structure analysis, the data quality for the guest structures in the crystalline sponge method is not simply determined by the crystal quality and X-ray experiments. We emphasize that the following issues are particularly important for obtaining high data quality: (1) smooth and complete solvent exchange in the pores, (2) high guest occupancy in the pores, (3) thorough thermodynamic equilibration of the absorbed guest, and (4) careful data collection particularly at high-angle regions. It is worth noting that tailored soaking conditions for every target guest should be designed based on the understanding of host–guest chemistry as well as on feedback from X-ray crystallographic experiments. As such, unlike for common X-ray crystal structure analysis, sophistication and craftsmanship are required not only in crystallography but also in host–guest and synthetic chemistry for successful analysis. The technique should be ideally performed (at least directed) by scientists who can survey these different fields. We recommend the guest soaking and crystal structure analysis be performed by the same researcher.

#### 2.1.3. Solvent exchange   

The pores of the as-synthesized crystalline sponge are filled with nitrobenzene, which is used as the solvent for the sponge preparation. Target guests are not efficiently absorbed into the as-synthesized sponge because nitrobenzene itself is a good guest with a high affinity to the pores. Therefore, replacing nitrobenzene with a non-interactive inert solvent is a crucial step. Typically, cyclohexane is used as the inert solvent. Pentane is useful when the guest soaking is examined at low temperature (∼ 0°C). The solvent exchanging process can be monitored by IR spectroscopy; Fig. S1 of the supporting information shows that the signal at 1346 cm^–1^, assignable to nitrobenzene, almost completely disappears after soaking in cyclohexane for 1 week at 50°C. The complete solvent exchange can be confirmed by elemental analysis or even by crystallographic analysis. Single-crystal X-ray analysis confirms the presence of ordered cyclohexane molecules in the pores, which indicates successful solvent exchange (Fig. S2). After completing the solvent exchange with cyclohexane, the crystalline sponge is ready to use. Clardy reported that the solvent exchange step can be omitted if the crystalline sponge is synthesized in chloroform–methanol (Ramadhar *et al.*, 2015*a* ▸,*b* ▸). This modified method gives a chloroform-filled crystalline sponge and is, therefore, probably effective only if the target compound is a better guest than chloroform or if a large excess of the target compound can be used.

The yield of high quality crystals suitable for the crystalline sponge method is actually not very high (< 5%) and, as in common single-crystal X-ray experiments, researchers often have to carefully choose the top quality crystals from many crystals under a microscope. It is rather an advantage of the crystalline sponge method that this quite inefficient step (crystal selection) can be undertaken before guest absorption. Namely, the required amount of the sample can be reduced to nano-to-microgram quantities by undertaking the crystal selection first and the guest soaking second. In this aspect, the crystalline sponge method is clearly distinguished from cocrystallization methods (Desiraju, 2007 ▸; Vishweshwar *et al.*, 2006 ▸) such as the pioneering work of Toda (1987 ▸) who co-crystallized a crystalline host with a non-crystalline guest using host–guest interactions. In this method, high quality crystals are selected at the last step and hence a substantial amount of sample is necessary to prepare the guest-cocrystallized single crystals.

#### 2.1.4. Guest soaking   

Any guest molecules that can interact with the sponge pores better than cyclohexane can be in principle absorbed. As cyclohexane is a poor solvent for common organic compounds, the minimum amount of a good solvent such as chloroform or dichloromethane is added to dissolve the guest in the guest-soaking step. Excess of the good solvent should be avoided because it will extract the target guest from the sponge crystal. The guest-soaking process is the most crucial step and usually determines whether the method works or not, or whether the data quality is good or bad. The following discussion describes how the soaking method has been improved and elaborated on. The history of the guest-soaking step almost parallels that of the crystalline sponge method itself.

**Figure 4:**
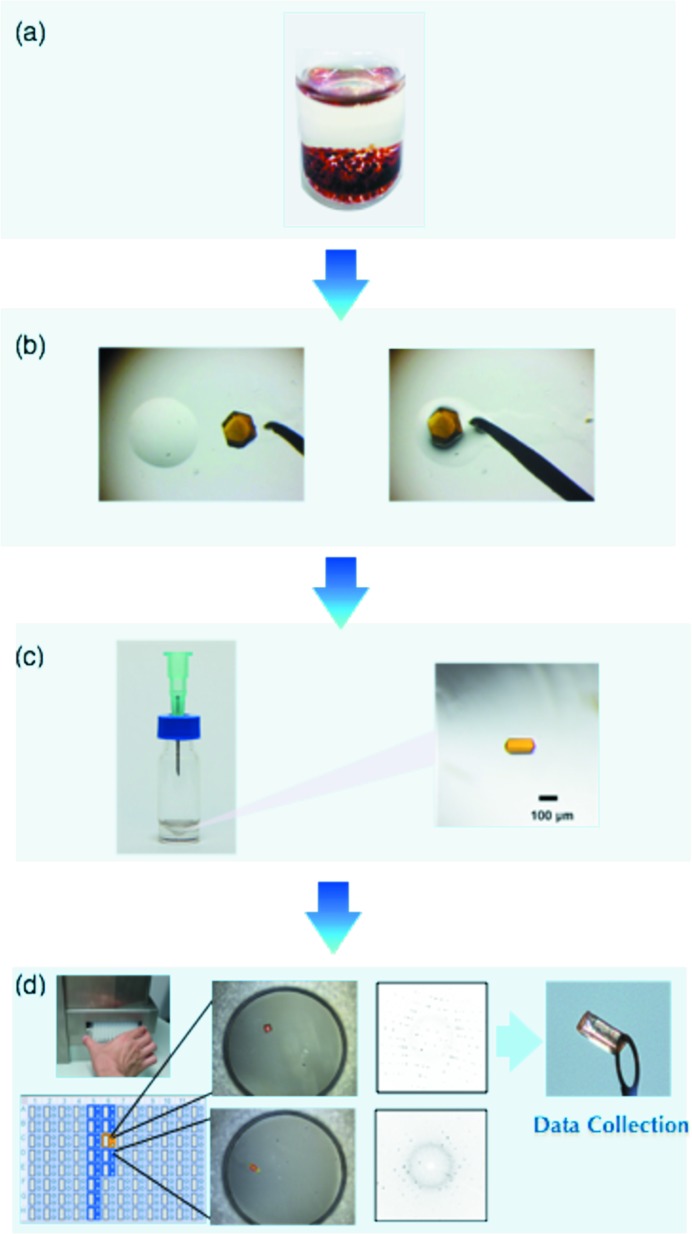
Evolution of the guest-soaking method. (*a*) Original method. (*b*) A-grain-of-crystal method. (*c*) Slow evaporation method. (*d*) High-throughput method.

1. (*i*) *Original method*: The prototype of the crystalline sponge method can be ascribed to the observation of single-crystal-to-single-crystal (SCSC) solvent exchange in the pores of porous complex (1), which we reported in 2002 (Biradha & Fujita, 2002 ▸). This facile solvent exchange occurs on simply dipping the crystals of (1) in the solvent. Similar SCSC exchange of solvents or guests in porous complexes have been reported by us and others (Kawano & Fujita, 2007 ▸; Suh *et al.*, 2002 ▸; Kawano & Fujita, 2007 ▸; Deiters *et al.*, 2005 ▸; Li *et al.*, 2009 ▸). Meanwhile, discrete *M*_6_ *L*_4_ cages that possess a cavity surrounded by the same tpt ligand used in (1) show remarkable guest binding properties in aqueous solution (Kusukawa & Fujita, 1998 ▸; Yoshizawa *et al.*, 2009 ▸). Inspired by the highly efficient guest binding by the discrete cage, we examined guest uptake by the crystals of (1) and in 2004 demonstrated the SCSC absorption of large organic molecules, such as pyrene, perylene and triphenylphosphine oxide, from their solutions (Ohmori *et al.*, 2004 ▸, 2005 ▸). These results convinced us of the strong guest-binding ability of the tpt-surrounded cavity and prompted us to use this phenomenon for structural analysis. In these prototype crystalline sponge methods, a substantial amount of the host complex (typically ∼ 10 mg; 1000–10000 crystals) was used (Fig. 4 ▸ *a*). However, this original method is still useful and can be the method of choice when a large quantity of the target compound is available. There is no technical difficulty in this original method.
2. (*ii*) *A-grain-of-crystal method*: To establish the method for the structural analysis of small molecules, the required amount of the sample should be reduced. This is especially necessary when the target compounds are highly valuable and obtained only in minute quantities; this is the case when analyzing isolated natural products, metabolites and impurities in drugs and foods, or flavor components. Thus, we reduced the experimental scale of the original method to the extreme by performing the guest-soaking experiment with only one grain of the sponge crystal (Fig. 4 ▸ *b*). From the crude solvent-exchanged sponge crystals, a top quality crystal was chosen and contacted with a small amount (typically, a few milligrams) of neat liquid sample or a supersaturated viscous solution on a glass plate. The guest absorption is usually completed in minutes to hours, although depending on the samples and crystal size it can sometimes take a few days. It is noteworthy that the order of events in the original method (guest-soaking followed by crystal selection) is reversed in the ‘a-grain-of-crystal’ method (namely, crystal selection followed by guest soaking), and this reversed protocol made it possible to analyze very small amounts of the target compounds. The method itself is technically not particularly difficult because there are only a limited number of parameters that influence the data quality.
3. (*iii*) *Slow evaporation method*: In the above method, the quality of the crystals often deteriorates severely during guest absorption if the host–guest interaction is too strong and the host framework is mechanically stressed. To perform the guest absorption with [(ZnI_2_)_3_(tpt)_2_·*x*(solvent)]_*n*_ (**1**) under much milder conditions, the crystals can be soaked in a diluted solution of the guest (Fig. 4 ▸ *c*). The use of a poor solvent is essential because otherwise the guest is extracted from the crystal; cyclohexane with a minimum amount of CH_2_Cl_2_ is typically used as the solvent. In this method, the sponge crystal is placed in a vial and soaked in the solution of the guest. The vial is equipped with a needle, through which the solvent slowly evaporates. During this slow evaporation, the guest is gradually concentrated, finally reaching the saturation point, and is forced to diffuse into the crystal. The data quality in the crystalline sponge method depends highly on this guest-soaking step. As emphasized in the general discussion of the standard protocols, there is no all-purpose protocol for this soaking step that can be applied to all compounds. The guest-soaking time ranges from minutes to days, or sometimes even weeks.Typically, a set of several conditions are examined. The parameters include soaking temperature, soaking time, solvent, concentration, crystal size, evaporation rate (controllable by changing the thickness of the needle). Guest absorption is, in general, faster at higher temperatures but the guest seems to be more concentrated and ordered in the crystal after soaking at lower temperatures. Annealing of the crystals during the guest-soaking is sometimes effective (*e.g.* 50°C, 2 d → room temperature, 2 d → 4°C, 2 d). The guest concentration in the crystal can be roughly estimated by microscopic IR measurement and this helps in the optimization of the soaking conditions. Although this slow evaporation method has considerably expanded the scope of the crystalline sponge method, it is technically difficult because every step in the method is unfamiliar and the optimization of the soaking conditions requires experience. We recommend that those new to the crystalline sponge method experience the guest-soaking techniques in the order described here [first (i)[Other l1li1], then (ii)[Other l1li2] and finally (iii)[Other l1li3]]. A benchmark test discussed later may help researchers to evaluate their level of expertise with the method.
4. (*iv*) *High-throughput method*: If satisfactory data are not obtained under a few standard conditions, other soaking conditions are examined. The soaking conditions can be optimized by using a high-throughput method, in which dozens of vials, each containing a sponge crystal, are used. For the guest-soaking, *m* different conditions (typically, *m* = 5–10) are applied, for each of which *n* sponge crystals (typically, *n* = 3–5) are used. Namely, *m* × *n* sponge crystals are subjected to guest soaking at once. The *m* × *n* guest-absorbed sponge crystals are transferred to a 96-well cell plate and screened by an X-ray scanner (Fig. 4 ▸ *d*). Every scan takes approximately 5 min and all the crystals are examined in 1–2 h. Cracked crystals are removed before the screening. Crystals with weak or spelt diffraction are visually checked and eliminated. Small invisible cracks and inhomogeneous inclusion of guests lead to large mosaicity. The best-diffracting crystal that shows sharp spots with the highest resolution is then selected for further study. This high-throughput method considerably shortens the trial-and-error process for optimization of the soaking conditions and is quite effective when representative standard conditions do not work well.
5. (*v*) *Other methods*: Some variations of these guest-soaking methods are still being developed. Like in protein ligand-soaking experiments, the use of micrometer-sized crystals (10–20 µm in size) has shown some excellent advantages over the standard method, which uses 100–200 µm sized crystals (micro-crystal method). For example, nanogram quantities of samples can be measured with faster and more homogeneous guest absorption, and without deterioration in the crystals. The details of the micro-crystal method will be discussed in a future report.

### 2.2. Crystallographic analysis   

#### 2.2.1. Guest occupancy   

One of the most important parameters that determine the data quality is the occupancy of the guest. The occupancy of the guest can be roughly estimated by microscopic IR spectrometry. Fig. S3 shows the relationship between soaking time (or guest site-occupancy) and *F*_o_ map for the inclusion of (5) in **1**. In one of our trials, the amount of absorbed guest after 2 d of soaking time was low and we could not clearly observe the guest electron density (Fig. S3*a*). By simply extending the soaking time to 7 d, however, the guest electron density was clearly observed after least-squares treatment of the host framework (Fig. S3*b*). By using a large excess of the guest, the guest site occupancy after 7 d reached almost 100%, and we could obtain better data for (5) (Fig. S3*c*).

Since the soaking time is not a highly reproducible parameter (because it depends on crystal size that cannot be precisely regulated), we can discuss only the following tendency from our observations: At low concentrations, ordering of the guests in the crystal is difficult because they cannot interact with each other. However, above a certain concentration, guest molecules seem to start to order in the crystal. In this regard, we suggest that the best description for the principle of the crystalline sponge method is ‘crystallization within crystals’ or ‘post-crystallization’.

#### 2.2.2. Molecular recognition sites   

**Figure 5:**
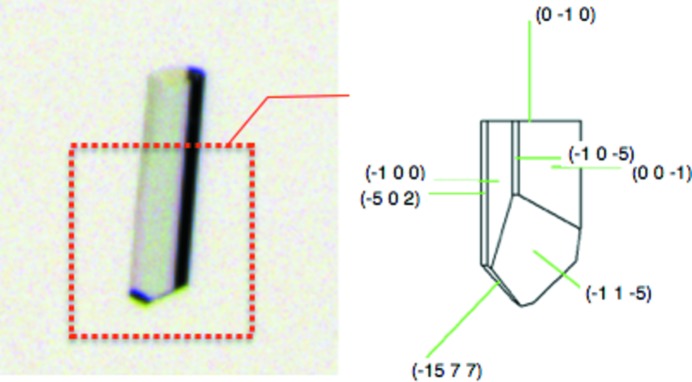
Face indexes of **1**. The left photo is **1** on a microscope. The crystal with face indexes described on the right was obtained from part of a crystal enclosed by the red broken line.

Two channels (*a* and *b*) are found in the views along with [010] and [101] directions, respectively, in the sponge crystal (Figs. 1 ▸ *b* and *c*). The faces indexed as (010) and (101) are not surfaces of a spontaneously grown crystal of **1**. However, (010) is considered as a cleavage face because a surface indexed as (010) is found in many split crystals. A typical crystal of **1** with face indexes is shown in Fig. 5 ▸.

**Figure 6:**
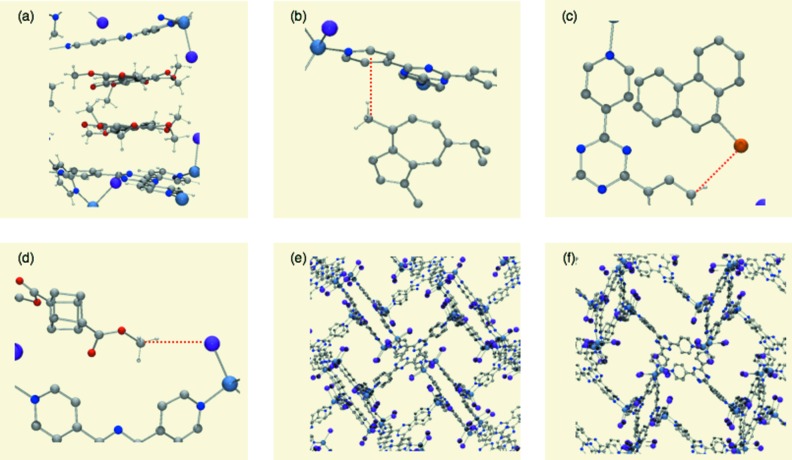
Various guest binding modes found in the pore of the crystalline sponge. (*a*) Donor–acceptor aromatic stacking between the nobiletin (**5**) guest and the tpt ligand. (*b*) CH–π interaction between methyl and pyridyl groups found in the binding of guaiazulene (**3**). (*c*) Hydrogen-bonding interaction with the bromine atom of bromophenthrene (**12**). (*d*) Hydrogen-bonding interaction of the methyl group of cubane (**11**) with the iodine atom of ZnI_2_. (*e*, *f*) Induced-fit deformation of the host framework: (*e*) before and (*f*) after the inclusion of tangeretin (**6**). The guest is omitted for clarity.

As rod-like molecules often lie along the channel *b*, most guest compounds seem to penetrate into the pore *b* from the (101) face of the crystal. Several different sites and modes of guest binding are observed in the pores of the crystalline sponge (Fig. 6 ▸). The most common feature of the guest binding is the efficient host–guest stacking on an electron-deficient tpt ligand. A typical example is the binding of **5** (Fig. 6 ▸ *a*). The pyridyl protons on the tpt ligand are good hydrogen-bond donors and are often located within a hydrogen-bond distance from the nitrogen and oxygen lone pairs of the substrates (Fig. 6 ▸ *b*). Even the Br atom of the guest can be a hydrogen-bond acceptor (Fig. 6 ▸ *c*). The iodine atom of the ZnI_2_ center seems to be a good hydrogen-bond acceptor (Fig. 6 ▸ *d*). The host network is relatively flexible and is quite often deformed before and after the guest absorption, which results in efficient induced-fit molecular recognition (Figs. 6 ▸ *e* and *f*).

When the sponge crystal is soaked in a guest solution, the guest molecules can freely diffuse into the solvent-filled pores and find the most suitable binding sites *via* reversible host–guest complexation. The binding sites in the pores vary case-by-case and are unpredictable for any guest. When a large association constant is gained at the binding site, the guest is trapped and concentrated. In other words, finding at least one suitable strong binding site is essential for every guest to be ordered and crystallographically analyzed.

#### 2.2.3. A benchmark experiment   

Although the details of the structural analysis of the guest-soaked crystalline sponge vary case-by-case depending on the guest examined, common and general methods for the analysis can be suggested. Taking guaiazulene as an example, a general procedure for the analysis is described. We recommend that the data for guaiazulene be used as a benchmark for evaluating guest soaking and crystal structure analysis in the crystalline sponge method. Another benchmark test for this method toward a chiral molecule is also proposed with a santonin guest. The obtained crystallographic data represents the potential ability of this method for reliable absolute structure determination.

#### 2.2.4. X-ray diffraction data collection   

Cyclohexane-filled crystalline sponge **1** is labile and rapidly deteriorates when it is taken out of the cyclohexane solvent because the solvent is lost from the pores by rapid evaporation. Upon inclusion of non-volatile guests, however, the crystals become relatively stable and can be treated with various protectants. After coating with an appropriate protectant, the guest-absorbed crystal is subjected to data collection. For the guaiazulene-containing crystals, we used Hampton Type A low viscosity oil as a protectant.

Several different X-ray source/detector combinations have been examined for the X-ray diffraction data collection. Data collection with a sufficient number of reflections and a suitable *I*/σ value at the high-angle region (*d* < 1.0) is particularly important to obtain high data quality for the trapped guest in the crystalline sponge as reflections from a guest is much weaker than those from the host at high-angle region. Although Mo *K*α radiation is commonly used for the X-ray structure analysis of metal complexes because of the weaker X-ray absorption by heavy atoms, Cu *K*α radiation has provided, overall, better results in the crystalline sponge method. The crystalline sponge is a weakly diffracting crystal because of the huge number of solvent molecules (typically cyclohexane) in the pores. Thus, the value of *I*/σ at the high-angle region by the Mo *K*α radiation is typically 1 or lower. However, Cu *K*α radiation provides a much higher *I*/σ value for the high-angle region. For guaiazulene, an exposure time of 90 s per data frame was applied for the high-angle region to satisfy the condition *I*/σ > 10. Under these conditions, high-resolution data were collected for guaiazulene with sufficient intensity, and the 0.82–0.79 Å shell exhibited a completeness of ∼ 90%. More than 1 d (27 h) was required for complete collection of the above data.

Using a synchrotron X-ray source (Ramadhar *et al.*, 2015*a* ▸,*b* ▸) is expected to shorten the data collection time. Synchrotron data may improve resolution for a given crystal, but the associated practical problems (*etc.*) mean that it may be less effective than a good in-house facility. Prompt feedbacks from the crystal structure analysis to the soaking experiments are very important to optimize the soaking conditions. Also, beam-times for experiments at a synchrotron facility are usually limited, and highly experienced operations are necessary to find the suitable conditions for data collection (*e.g.* wavelength, detector, attenuator, data scan strategy; see Table S1). All the data in Table 1 ▸ were collected with an in-house X-ray diffractometer with a Cu *K*α radiation source.

#### 2.2.5. Space group determination   

The crystalline sponge has the centrosymmetric *C*2/*c* symmetry. This crystallographic symmetry is maintained after inclusion of an achiral guest in many cases. In the case of inclusion of a chiral guest, this space group changes to a non-centrosymmetric one (*C*2) but geometrical distortion in the framework is not obvious. This slight change of framework causes a pseudo-symmetry issue (faint *C*2/*c* symmetry) and may lead to misassignment of the space group. The space group of the guest-included crystalline sponge should be carefully determined based on the extinction rule without excessively relying on an automatic suggestion by a space-group determination program.

#### 2.2.6. Refinement of the framework   

After obtaining an initial structure for refinement using a standard program [*SHELXT*, (Sheldrick, 2015 ▸), *SHELXS* (Sheldrick, 2008 ▸), *SIR* (Burla *et al.*, 2015 ▸), or *SUPERFLIP* (Palatinus & Chapuis, 2007 ▸)], the refinement of the host framework should be performed first. After anisotropic refinement of all non-H atoms in the framework, relatively high residual electron-density peaks are found around zinc and iodine atoms. Since the ZnI_2_ fragments are the most flexible part of the network, disordered typically two ZnI_2_ fragments can usually be modeled and properly refined anisotropically (Fig. S4). After this disorder treatment on the ZnI_2_ fragments, a partial or full guest molecule clearly appears in the difference electron density map. In the analysis of the guaiazulene-absorbed crystalline sponge, the initial structure was obtained as described above.

#### 2.2.7. Refinement of the guest   

**Figure 7:**
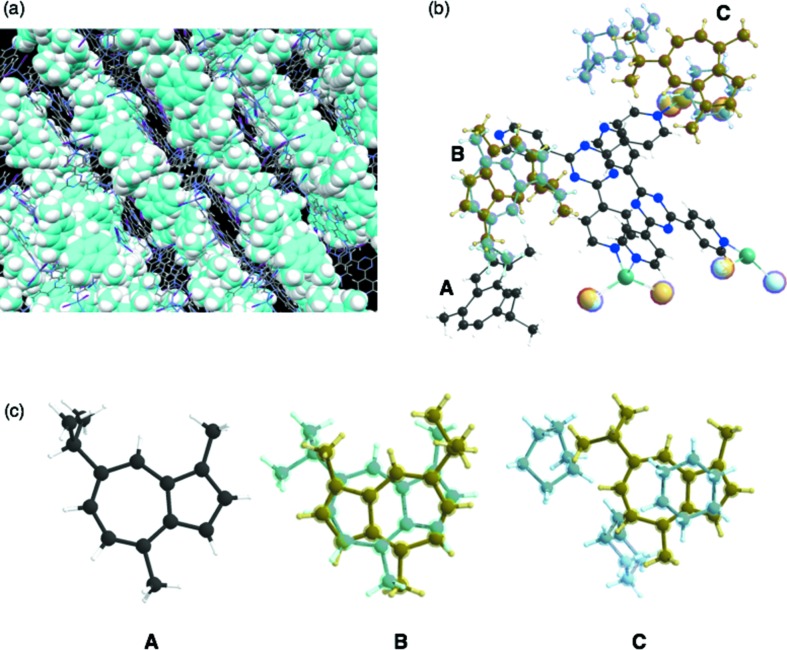
Crystal structure of the guest absorbed crystalline sponge (guest = guaiazulene, **3**). (*a*) The three-dimensional network structure (a projection down [010]). The host and guest are represented by stick and space-filling models, respectively. Solvents filling the voids are omitted for clarity. (*b*) Asymmetric unit structure. Three independent guaiazulene molecules (**A**–**C**), along with cyclohexane solvents are observed. Molecule **A**: observed with s.o.f. = 1 without disorder; **B**: fourfold disordered around the twofold axis with the two statistically disordered guaiazulene molecules [s.o.f. = 0.278 (10) and 0.222 (10), respectively]; **C**: disordered with solvents with s.o.f. = 0.186 (6). The superimposed blue and yellow colors on some atoms show the use of a PART command and represent the disordered model. (*c*) The guests **A**–**C** (and overlapping cyclohexane molecules) are individually shown.

If a guest molecule is placed on a general position and its site occupancy factor (s.o.f.) is almost 1, the guest structure can be properly refined using a typical refinement procedure. In the case of the guaiazulene-absorbed crystalline sponge, one guaiazulene molecule (**A**) in the asymmetric unit could be refined with 100% occupancy (s.o.f. = 1; Fig. 7 ▸).

If the guest is located on a symmetry element (*i.e.* a special position) of the framework, the independent guest molecule and a symmetrically generated one are crystallographically equivalent. Therefore, the occupancy of the guest should be divided by the multiplicity of the special position during the refinement. In the guaiazulene case, the second independent guaiazulene molecule (**B**) was refined using a disordered model of two orientationally different guests with the constraint of the sum of their occupancy being 50% because it was placed on a twofold axis (multiplicity = 2). The refined s.o.f.s for both independent guests are 0.278 (10) and 0.222 (10). This means that the total occupancy of the guest in this site is 100%.

If the guest occupancy is considerably lower than 100%, guest molecules should be overlapped with solvent cyclohexane molecules and refined using a disordered model of the guest and cyclohexane. The structure of the guest for refinement is made by selecting some residual electron density peaks in the difference electron density map. The s.o.f. of the guest should be constrained temporarily to a reduced value (typically, 0.3–0.5) to find the overlapped cyclohexane molecules. After refinement of the guest, the overlapped cyclohexane molecules are found as a difference electron density map. Geometries and s.o.f.s of the guest and the cyclohexane are refined using this disordered model. For guaiazulene, the s.o.f. of the third independent molecule (**C**) disordered with solvent was estimated as 0.186 (6) from least-squares refinements. Some restraints are applied in this refinement, and the sum of the s.o.f.s of the guest and solvent is constrained to 100% to refine the s.o.f.s of the guest and cyclohexane. When overlapped cyclohexane molecules are not found, we postulated s.o.f. = 1 to satisfy the 100% sum. In this case, some overestimation is unavoidable if the actual occupancy is slightly less than 100%.

#### 2.2.8. Refinement of remaining solvent molecules   

As mentioned above, filling the remaining void of the pores with inert solvent molecules (cyclohexane in the present case) is an important requirement for efficient guest inclusion. In the crystalline sponge method, less interactive solvents (hydrocarbons) have the advantage of being easily replaced with the guests, but have the disadvantage of being disordered when some solvent molecules remain after guest-soaking. Therefore, refinement of solvent molecules is still challenging in the crystalline sponge method. Unfortunately, the maximum resolution in the case of Cu *K*α radiation is not so high (*d* ≃ 0.8 in our cases) and the present data resolution cannot provide electron densities corresponding to cyclohexane molecules with atomic resolution: broad and obscure electron densities are found as an averaged structure of variously orientated cyclohexane molecules. The observation of unusual conformations (boat or twisted) in an averaged structure for some of the included solvents is unavoidable to some extent. In the original paper, these disordered solvent molecules were eliminated by using the *SQUEEZE* procedure (Spek, 2015 ▸) in the program *PLATON*. However, whether it is appropriate to apply such a solvent-masking procedure to the observed electron density is still a matter of debate (Spek, 2015 ▸). In the present study, all cyclohexane molecules were found and refined to avoid applying the *SQUEEZE* procedure. Before the guest soaking, 5.5 cyclohexane molecules were refined in the asymmetric unit. The disordered cyclohexane molecules in a guest-soaked crystalline sponge were also refined although some restraints were necessary. Refinement of cyclohexane molecules in the crystal improved the phases of reflections and helped to reduce the number of restraints needed for refinement of a guest structure. Therefore, refinement of guest and solvent molecules in parallel is highly recommended.

#### 2.2.9. Absolute structure determination   

One of the biggest advantages of the crystalline sponge method is the possibility for absolute structure determination of the absorbed guest molecule using anomalous scattering from the heavy atoms installed in the framework (Yoshioka *et al.*, 2015 ▸; Zigon *et al.*, 2015 ▸). Using this feature, the absolute configuration of a chiral molecule that possesses no heavy atoms can be determined in an efficient way. This would be the only direct method for determining the absolute structure of a trace amount or oily compound. Although current crystallography does not always need heavy atoms for the absolute structure determination (Fujita *et al.*, 1994 ▸; Parsons *et al.*, 2013 ▸; Hooft *et al.*, 2010 ▸) much higher quality data are required and, in principle, the reliability decreases without heavy atom effects. When the crystalline sponge method is used for absolute structure determination, however, we recommend that researchers follow the guidelines described below because there are some important warnings in applying this method.

The guidelines are discussed here along with a typical procedure for santonin (**4**), a classical anthelmintic drug, the absolute structure of which was a matter of discussions in the 1950s (Corey 1955 ▸). In our original paper, guest-soaking was carried out at 323 K for 2 d (our preliminary standard conditions). To obtain better diffraction data in this study we prepared four vials, each containing a sponge crystal (typically 200 × 100 × 80 µm^3^ in size) with 45 µL of cyclohexane. Then, 5 µL of the guest solution in CH_2_Cl_2_ (2.5 µg μL^−1^) was added to each vial. The vials were pierced with a needle for solvent evaporation and kept at 50°C. After 1 d at 50°C, the vials were kept at 4°C for a further 1 d to facilitate further guest inclusion. The most suitable crystal was selected by preliminary diffraction experiments on all the prepared crystals using an X-ray scanner. For data collection, the use of Cu *K*α radiation is again highly recommended for obtaining better *I*/σ values with a more pronounced intensity contrast between Bijvoet pairs.

Single-crystal-to-single-crystal framework deformation concomitant with symmetry lowering from *C*2/*c* to *C*2 or *P*2_1_ has been observed upon chiral guest inclusion in previous studies (Yoshioka *et al.*, 2015 ▸; Zigon *et al.*, 2015 ▸; Inokuma *et al.*, 2013 ▸). For santonin, the space group was changed from *C*2/*c* to *P*2_1_. This space group change was clearly indicated by the preserved extinction rule of 0*k*0 (*k* = odd number) and by the statistics of the normalized structure factor (〈|*E* ^2^ − 1|〉 = 0.806 in the present case). For some other chiral guests, the *C*2/*c* symmetry is lowered to *C*2. However, evaluation of centrosymmetry based on the statistics of normalized structure factor often failed because of the very small deformation resulting in the pseudo-symmetry of the host framework. Therefore, the appearance of *h*0*l* (*l* = odd number) reflections, which is evidence for the disappearance of the *c*-glide plane involving the inversion center, should be carefully confirmed. This pseudo-symmetry problem sometimes decreases the reliability of the absolute configuration determined by the crystalline sponge method. For example, when limonene was examined as a chiral guest, the guest molecule was found by crystal structure analysis, but the appearance of *h*0*l* (*l* = odd number) reflections could not be confirmed and the absolute configuration was obscured. One reason that the pseudo-symmetry problem occurred with limonene is presumably its low guest occupancy in the crystalline sponge and its pseudo-mirror symmetrical skeleton. To avoid the pseudo-symmetry problem, a high guest occupancy of above 50% is desired.

In the structure refinement, five independent santonin molecules were refined without using restraints or constraints (Table 1 ▸). The Flack parameter calculated using the Parsons’ method is −0.0071 (11), which clearly represents the definitive determination of the absolute configuration of santonin. We thus highly recommend santonin as a benchmark for the absolute structure analysis of chiral molecules.

**Figure 8:**
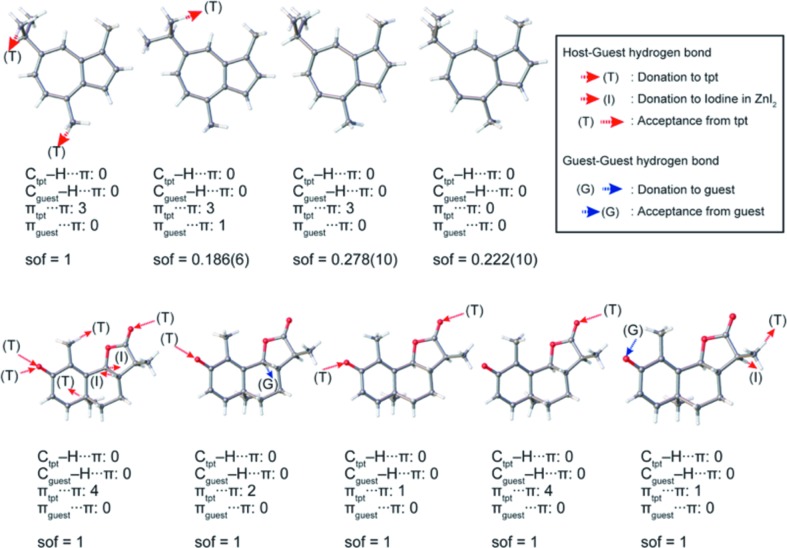
Intermolecular interactions involving guest molecules of guaiazulene and santonin in the crystalline sponge.

The reason why santonin induced significant crystallographic symmetry lowering from *C*2/*c* to *P*2_1_ is considered to be the presence of a relatively large number of host–guest interactions than other molecules. Intermolecular interactions involving guest molecules in **3** and **4** are shown in Fig. 8 ▸. The number of host–guest interactions in **4** (25 interactions) is more than double for **3** (12 interactions). Larger expansion of the pores by inclusion of santonin rather than guaiazulene (Figs. 2 ▸ *b* and *c*) indicates that a considerable number of interactions leads to significant distortion of the host framework and obvious space group change from achiral (*C*2/*c*) to chiral (*P*2_1_).

If another enantiomer is available for the target chiral compound, double checking of the absolute configuration by analyzing both enantiomers using the crystalline sponge method is strongly recommended. When the guest occupancy is low, often the Flack parameter does not significantly decrease and the value remains around 0.2, presumably because of the pseudo-symmetry of the guest-absorbed structure. In such cases, obtained absolute configuration should be considered only as reference data.

We do not recommend the use of *SQUEEZE* program in the absolute structure determination because the Flack value calculated both in a classical way or the Parsons’ method is based on the modeled structure in refinement. In particular, when the electron densities of an un-modeled chiral guest is anticipated in the void space, the Flack value after *SQUEEZE* treatment may be unsuitable because contribution of the anticipated guest is eliminated in the calculation.

#### 2.2.10. Details for other compounds   

(*i*) *Flavonoids*: Three flavonoid compounds [nobiletin (**5**), tangeritin (**6**), heptamethoxyflavone (HMF, **7**)] were analyzed with good data qualities (Table 1 ▸). In the flavonoid cases, the improvement in the data quality is to some extent due to the difference in sample purity. In our original paper, the purpose of the original experiments was to demonstrate a proof-of-concept for LC–SCD (liquid chromatography–single-crystal diffraction) analysis and to show potential applications for the crystalline sponge method in the structural determination of natural products. Therefore, all the X-ray experiments were examined with microgram quantities of natural products directly isolated from orange peel, which were contaminated with irremovable impurities. It is noteworthy that the biased electron density (for example, that for **5** shown in Fig. S3*b*) nevertheless gave an acceptable fit to the model, which was constructed based on the chemical knowledge that the compound had a flavone (4H-chromen-4-one) skeleton. Although crystallographically poor, structural information gained from the electron densities is of great help for natural product chemists, particularly when the structural information is supported by or combined with other measurements such as NMR and MS. In the present study, pure chemical compounds **5**–**7** purchased from a reagent company were used.

(*ii*) *Cubane*: The structure analysis of cubane (**11**) is worthy of comment. Despite the high guest occupancies in the four sites [s.o.f. = 1, 0.55 (3) + 0.45 (3); two guests are disordered, 0.5; lie on an inversion center (multiplicity = 2), and 0.646(17)] and the appropriate treatment of void-filling solvents, the structure analysis resulted in a seemingly worse *R*_1_ value (0.1296). The reason for this moderate *R*_1_ value is due to the significant one-dimensional streak-shaped residual electron densities around ZnI_2_ portions in the difference electron density map. These residual densities suggest severe one-dimensional disorder of these parts. In the reciprocal space, diffused scattering along the pore directions [namely, (010) and (101) directions] are found. These results suggest one-dimensional periodicity loss involving geometrical uncertainty of ZnI_2_ portions.

By applying data treatment as a twinning crystal, crystallographic data was improved (*R*_1_ reached 0.0530 and 0.0546 for each phase). Reconstructed precession images for **1** and **11** (Fig. S15) represented that no twinning was found in **1**. Guest-induced twinning might be considerable in the crystalline sponge method.

(*iii*) *Others*: In our original report, one of the authors attempted the structure analysis of six compounds (**8**–**13**) using the sponge method without any additional chemical information. Although scientifically not particularly important, this attempt at *ab initio* structure analysis did allow us to learn some limitations and warnings for the method. In the analysis of **8**, for example, we were unable to distinguish whether the compound was a phenol, an aniline or a toluene. By combination with MS data, however, an aniline structure was correctly elucidated. In the updated data, the observed C*sp* ^2^—N*sp* ^3^ bond length is 1.33 (3) Å, and is clearly discriminated from the C*sp* ^2^—C*sp* ^3^ bonds in the structure [1.57 (5) and 1.52 (4) Å].

For compounds **8**–**13**, the soaking conditions have not been fully optimized; however, acceptable data qualities have been obtained. Presumably, the data qualities can be improved by further optimizing the soaking conditions.

### 2.3. Structure analysis of an unknown molecule using the crystalline sponge method   

The crystalline sponge method is applicable to determine an unknown molecule in some cases. One actual example is the structure analysis of the unexpected plasticizer (Zigon *et al.*, 2015 ▸). The synthesized compound is sometimes polluted by contamination of plasticizer molecules. In the previously reported crystal structure of a guest-included crystalline sponge (CCDC: 1053228), bis(2-ethylhexyl)phthalate, a kind of plasticizer, was properly modeled based on the distances of two non-hydrogen atoms and refined. The amount of this molecule in the synthesized oily guest compound was too low to detect by NMR analysis. However, the crystalline sponge method clearly shows this unexpected molecule in blind analysis.

## 3. Conclusion   

The crystalline sponge method has been updated to provide a reliable technique for the X-ray structure analysis of non-crystalline compounds in minute quantities. Unoptimized protocols in every step of the method, reported in our original paper, have been thoroughly optimized to be one of an applied technique in X-ray crystallography. The application of the crystalline sponge method in various molecular-based research fields is now highly anticipated.

The full optimization of all the steps for every compound is however not always easy. For practical reasons, also depending on the purpose, researchers may have to treat unoptimized data. In such cases, researchers should not forget the important major premise that *the crystalline sponge method is a part of crystallography and the supporting spectroscopic data (NMR and MS) would be helpful for determining the three-dimensional structure of target compounds in general crystallographic structure analysis.* Expected advantages of this method are (i) determination of the absolute structure of a molecule consisting of only light atoms and (ii) direct observation of the molecular structure of an oily compound. The crystalline sponge method would effectively work for those purposes. We also note that the protocols discussed here are still tentative and should be elaborated through future discussions among users and by many successful results.

The crystallographic analysis of ‘post-crystallized’ compounds in the pores with low site-occupancies is quite an unusual situation, and one that past crystallographers have seldom experienced. Researchers may encounter crystallographic phenomena or problems, including space-group changes, the formation of superlattice structure, pseudo-symmetry problems, treatment of guests lying on symmetry elements, and treatment of guests with low site occupancies, all of which were raised upon guest inclusion into the sponge crystal and may be unfamiliar and unexpected to chemists. In addition, the treatment of solvents that fill the void space is a common yet tedious problem in MOF structure analysis (Singharoy *et al.*, 2015 ▸). We suggest that researchers undertake all the crystallographic steps from data collection to structure refinement with great care, through collaboration with highly experienced crystallographers.

## Supplementary Material

Crystal structure: contains datablock(s) cyclohexane, guaiazulene, santonin, nobiletin, tangeretin, heptamethoxyflavone, diisopropylaniline, nitrobenzaldehyde, cinnamaldehyde, Dimethyl_cubanedicarboxylate, cubane_twin_1, cubane_twin_2, bromophenanthrene, vanillin. DOI: 10.1107/S2052252515024379/de5035sup1.cif

Structure factors: contains datablock(s) Dimethyl_cubanedicarboxylate. DOI: 10.1107/S2052252515024379/de5035Dimethyl_cubanedicarboxylatesup2.hkl

Structure factors: contains datablock(s) cyclohexane. DOI: 10.1107/S2052252515024379/de5035cyclohexanesup3.hkl

Structure factors: contains datablock(s) guaiazulene. DOI: 10.1107/S2052252515024379/de5035guaiazulenesup4.hkl

Structure factors: contains datablock(s) santonin. DOI: 10.1107/S2052252515024379/de5035santoninsup5.hkl

Structure factors: contains datablock(s) nobiletin. DOI: 10.1107/S2052252515024379/de5035nobiletinsup6.hkl

Structure factors: contains datablock(s) tangeretin. DOI: 10.1107/S2052252515024379/de5035tangeretinsup7.hkl

Structure factors: contains datablock(s) heptamethoxyflavone. DOI: 10.1107/S2052252515024379/de5035heptamethoxyflavonesup8.hkl

Structure factors: contains datablock(s) diisopropylaniline. DOI: 10.1107/S2052252515024379/de5035diisopropylanilinesup9.hkl

Structure factors: contains datablock(s) nitrobenzaldehyde. DOI: 10.1107/S2052252515024379/de5035nitrobenzaldehydesup10.hkl

Structure factors: contains datablock(s) cinnamaldehyde. DOI: 10.1107/S2052252515024379/de5035cinnamaldehydesup11.hkl

Structure factors: contains datablock(s) cubane_twin_1. DOI: 10.1107/S2052252515024379/de5035cubane_twin_1sup12.hkl

Structure factors: contains datablock(s) cubane_twin_2. DOI: 10.1107/S2052252515024379/de5035cubane_twin_2sup13.hkl

Structure factors: contains datablock(s) bromophenanthrene. DOI: 10.1107/S2052252515024379/de5035bromophenanthrenesup14.hkl

Structure factors: contains datablock(s) vanillin. DOI: 10.1107/S2052252515024379/de5035vanillinsup15.hkl

CCDC references: 1418972, 1418974, 1418979, 1418978, 1418980, 1418976, 1418973, 1418977, 1418970, 1418971, 1442316, 1442317, 1418969, 1418981

## Figures and Tables

**Table 1:** Molecular structures of guests **3**–**12** determined by the crystalline sponge method; our original data and the updated data are compared

			Data quality†	
Guest compound	*F*_o_ map‡	*ORTEP* §	This work	Original work¶
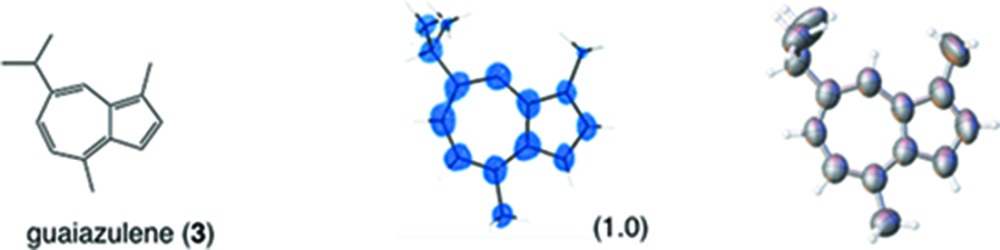			(1) 0.0279	0.0424
			(2) 0.0379	0.0859
			(3) 0.1035	0.3021
			(4) No	Yes
			(5) 1.056	1.097
			(6) 0	71
			(7) ∼ 100%	∼ 60%††
				
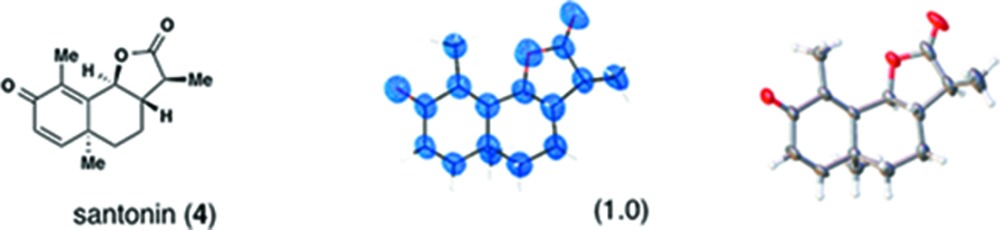			(1) 0.0421	0.0511
			(2) 0.0312	0.0827
			(3) 0.0781	0.1813
			(4) No	Yes
			(5) 1.020	1.101
			(6) 0	120
			(7) ∼ 100%	∼ 100%
			(8) −0.0071 (11)	0.092 (18)‡‡
				
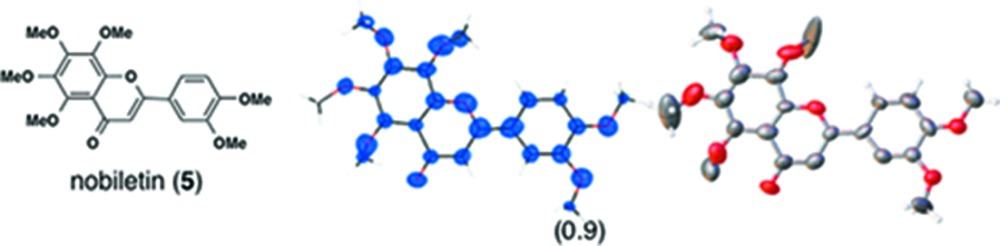			(1) 0.0332	0.0491
			(2) 0.0808	0.1065
			(3) 0.2136	0.2915
			(4) No	Yes
			(5) 1.197	1.052
			(6) 5	224
			(7) ∼ 51%	∼ 50%
				
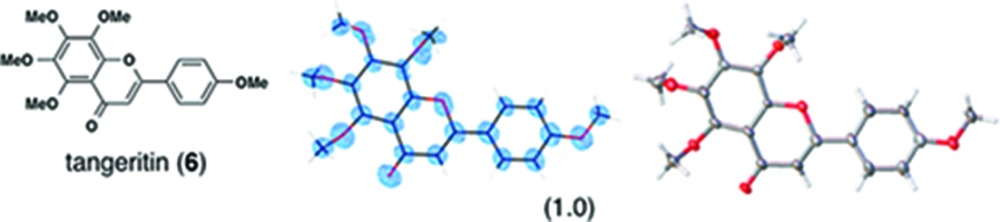			(1) 0.0701	0.0400
			(2) 0.0768	0.0823
			(3) 0.1730	0.2283
			(4) No	Yes
			(5) 1.091	1.043
			(6) 9	342
			(7) ∼ 100%	∼ 94%
				
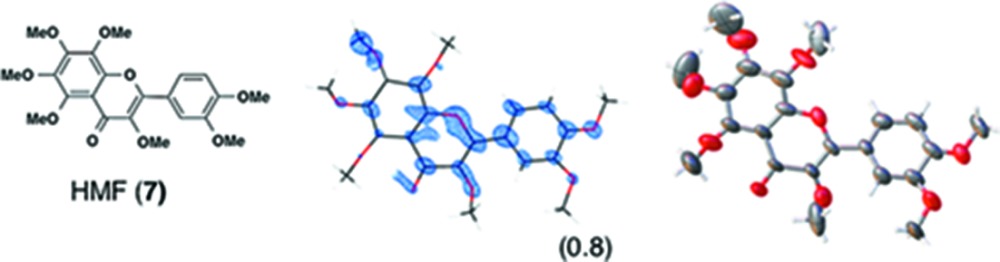			(1) 0.0492	0.073
			(2) 0.0536	0.073
			(3) 0.1545	0.2184
			(4) No	Yes
			(5) 1.033	0.893
			(6) 224	102
			(7) ∼ 60%	∼ 50%
				
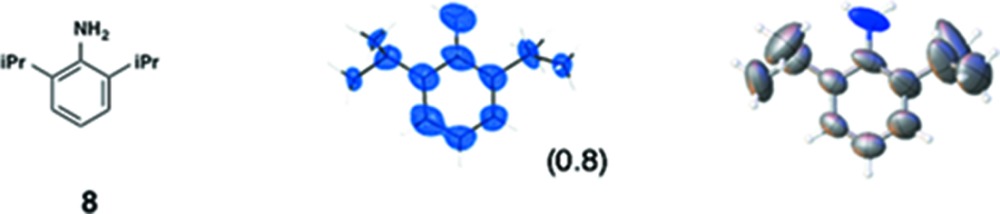			(1) 0.0309	0.0459
			(2) 0.0653	0.1182
			(3) 0.1541	0.3520
			(4) No	Yes
			(5) 1.128	1.082
			(6) 78	64
			(7) ∼ 100%	∼ 75%
				
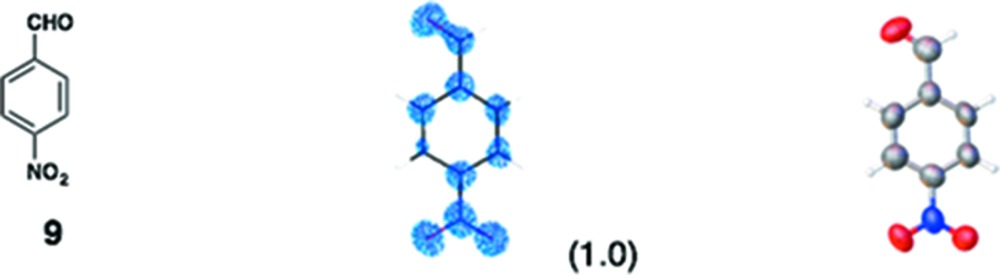			(1) 0.0342	0.0700
			(2) 0.0667	0.1130
			(3) 0.2061	0.3380
			(4) No	Yes
			(5) 1.090	1.217
			(6) 0	49
			(7) ∼ 100%	∼ 50%
				
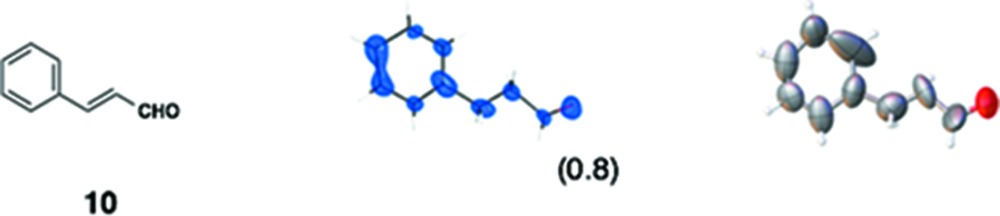			(1) 0.0305	0.0558
			(2) 0.0416	0.0750
			(3) 0.1216	0.2631
			(4) No	Yes
			(5) 1.044	1.173
			(6) 27	45
			(7) ∼ 100%	∼ 50%
				
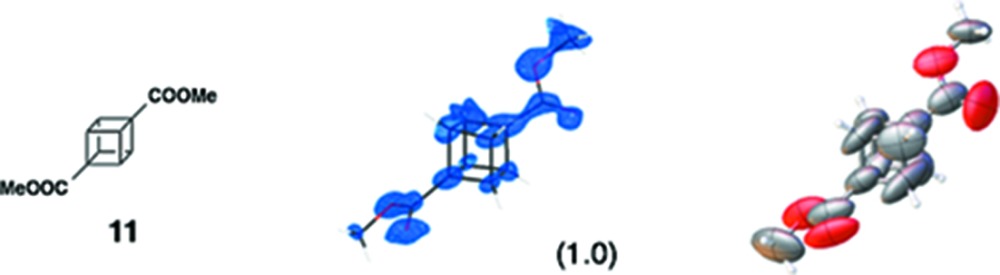			(1) 0.0605	0.1216
			(2) 0.1296	0.1345
			(3) 0.4390	0.3775
			(4) No	Yes
			(5) 1.574	1.082
			(6) 18	58
			(7) ∼ 100%	∼ 70%
				
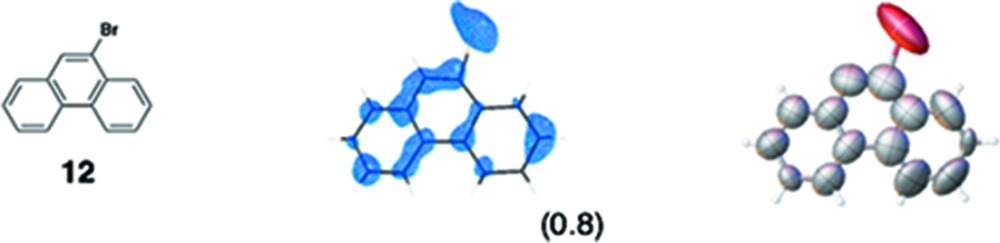			(1) 0.0343	0.0705
			(2) 0.0752	0.1162
			(3) 0.2359	0.3753
			(4) No	Yes
			(5) 1.038	1.448
			(6) 109	136
			(7) ∼ 63%	∼ 68%
				
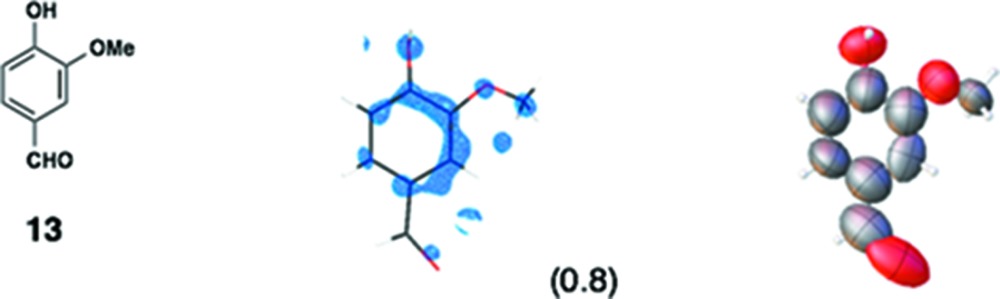			(1) 0.0450	0.0555
			(2) 0.0733	0.1103
			(3) 0.2397	0.3402
			(4) No	Yes
			(5) 1.038	1.158
			(6) 92	9
			(7) ∼ 100%	∼ 100%

† Data listed: (1) *R*_int_; (2)*R*_1_ [*F* ^2^> 2σ(*F* ^2^)]; (3): *wR*_2_; (4) *SQUEEZE* treatment; (5) GoF; (6) number of restraints (for the best-resolved guest); (7) occupancy (for the best-resolved guest); (8) Flack (χ) parameter (Flack value calculated by the Parsons’ method).

‡ Values in the parentheses are σ level.

§ 30% probability.

¶ Refinement was performed using *SHELXL*97 (Sheldrick, 2008 ▸) program.

†† Only 500 ng of the guest was used to examine the lowest limit of the sample amount required.

‡‡ Classic Flack parameter.
